# Autoimmune Hemolytic Anemia Following Uncomplicated Spinal Surgery: A Report and Brief Review

**DOI:** 10.7759/cureus.35591

**Published:** 2023-02-28

**Authors:** Westin M Yu, Hiren N Patel

**Affiliations:** 1 Department of Neurological Surgery, Lake Erie College of Osteopathic Medicine, Erie, USA; 2 Department of Neurosurgery, Massachusetts General Hospital, Boston, USA

**Keywords:** lumbar radicular pain, direct coombs test, interbody device, revision spinal fusion, anemia and hyperbilirubinemia, warm autoimmune hemolytic anemia, transforaminal lumbar interbody fusion (tlif), coomb's positive hemolytic anaemia, autoimmune hemolytic anemia (aiha)

## Abstract

This report and literature review describes a case of a Coombs test-positive warm antibody autoimmune hemolytic anemia (AIHA) in a patient following routine spinal surgery without complications. This is the first reported case of symptomatic direct Coombs test-positive warm antibody AIHA developing in a neurosurgical patient.

The patient is a 73-year-old female with left radicular leg pain who developed warm antibody AIHA following standard uncomplicated spinal surgery. A positive direct Coombs test confirmed the diagnosis in combination with characteristic laboratory values. The patient did not have any significant predisposing risk factors. On postoperative day (POD) 23, she presented with fatigue and characteristic laboratory values of decreased hemoglobin, elevated bilirubin, lactate dehydrogenase, and decreased haptoglobin. Hematology initiated and monitored appropriate treatment and proposed that the working hematologic diagnosis is stress-induced AIHA secondary to recent spinal surgery. The patient recovered well from a neurosurgical perspective and reported no neurosurgical complaints during the last follow-up.

A female presenting with left radicular leg pain developed symptomatic anemia following uncomplicated spinal surgery. A positive direct Coombs test in combination with characteristic laboratory values confirmed the diagnosis of warm antibody AIHA.

## Introduction

Autoimmune hemolytic anemia (AIHA) is a rare autoimmune disease that leads to hemolysis from the formation of autoantibodies and is classified as a type II hypersensitivity reaction. AIHA has an incidence of 0.8 to 3 people per 100,000 and a mortality rate of 11% [1]. AIHA can be classified as primary AIHA, which has an idiopathic etiology, or secondary AIHA. Secondary AIHA can present with other autoimmune diseases, including systemic lupus erythematosus (SLE), rheumatoid arthritis (RA), and others. Many infections, such as babesiosis and infectious mononucleosis due to cytomegalovirus (CMV), have also been associated with AIHA. AIHA has also been linked to many hematological malignancies, most notably chronic lymphocytic leukemia (CLL) [1-4]. A variety of medications have also been reported to cause AIHA, including penicillin and methyldopa. AIHA is further classified into a warm, cold, or mixed variant based on the temperature at which hemolysis occurs [1,2]. Surgery is not a known direct trigger for AIHA, and there has only been one other documented case of surgery-induced AIHA. The other documented case presents a patient developing AIHA after trauma surgery and subsequent sepsis. Surgery and the subsequent healing process can give rise to an increased state of stress and inflammation, leading to the development of an autoimmune disease. AIHA should be included in the diagnostic workup of an anemic patient following surgery once obvious etiologies have been considered. We report the first case of a neurosurgical-induced warm antibody AIHA.

## Case presentation

A 73-year-old Caucasian female presented to the neurosurgical office with complaints of radicular left leg pain along with characteristic dermatome findings. She had a previous relevant medical history of well-controlled beta thalassemia minor for two years. The patient's baseline hemoglobin level was approximately 10 g/dL due to her beta thalassemia minor diagnosis.

**Figure 1:**
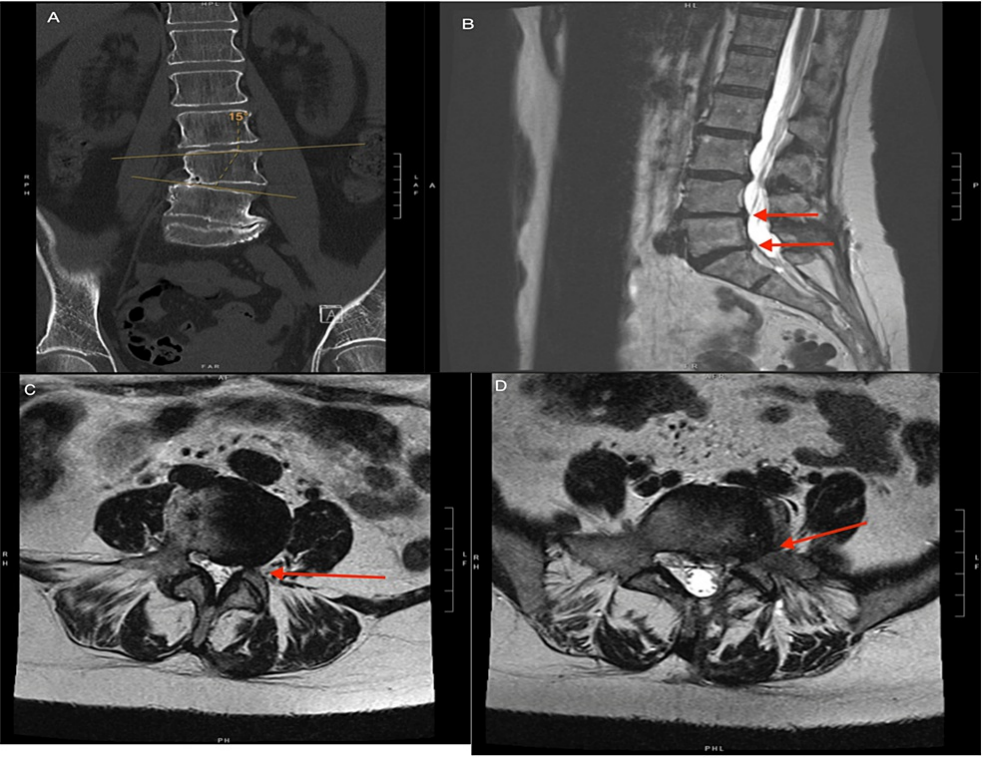
Preoperative Imaging: (A) coronal lumbar spine CT showcasing levoscoliosis measuring 15 degrees at L4-L5 with lateral listhesis to the left; (B) sagittal lumbar spine MRI showcasing L4/L5/S1 collapsed disc (red arrows) with degenerative disc disease and loss of disc height; (C) axial lumbar spine MRI showcasing L4/L5 left foraminal stenosis (red arrow); (D) axial lumbar spine MRI showcasing severe L5/S1 foraminal stenosis (red arrow). CT, computed tomography; MRI, magnetic resonance imaging

**Figure 2:**
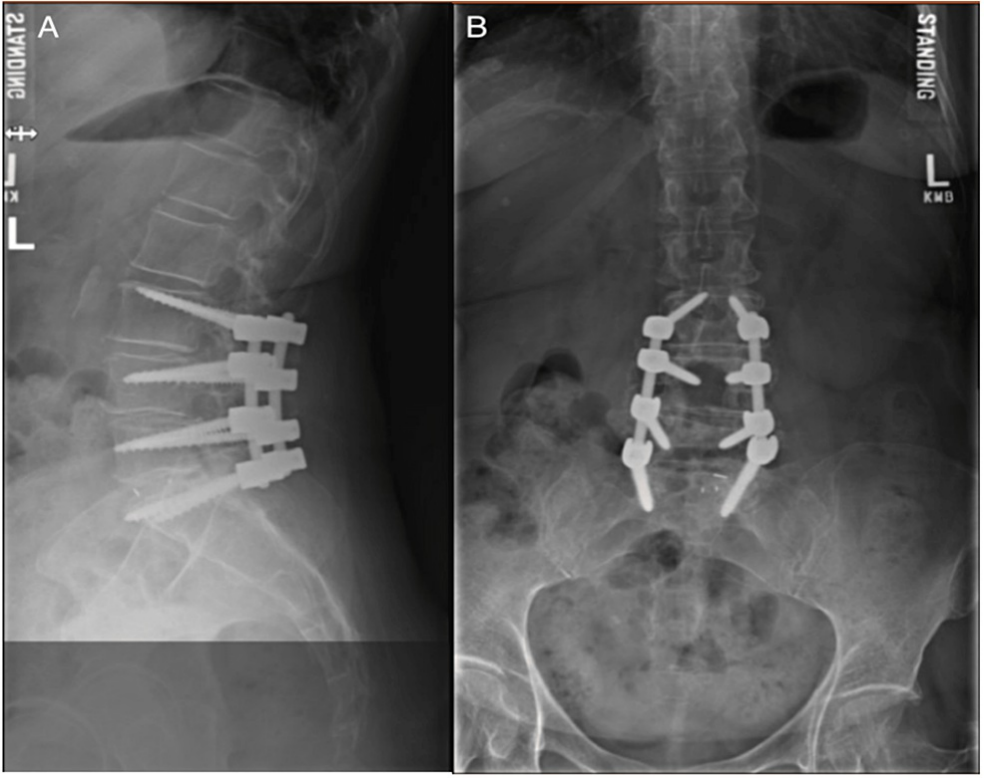
Postoperative X-ray: (A) sagittal standing lumbar X-ray showcasing the implanted spinal hardware; (B) coronal standing lumbar X-ray showcasing implanted spinal hardware.

The patient previously underwent a left L5-S1 hemilaminectomy and microdiscectomy performed four months prior due to radicular left leg pain in the S1 distribution with associated numbness and tingling in the same dermatomes. She initially had a resolution of symptoms and an uneventful recovery until the return of symptoms. Three months later, the patient presented with a recurrence of symptoms, with a physical exam demonstrating radicular left leg pain in the L5-S1 distribution with associated numbness and tingling and no other neurologic deficits. CT and MRI demonstrated an L5/S1 collapsed disc with severe left L5/S1 foraminal stenosis along with known L4-L5 coronal levoscoliosis measuring 15 degrees at L4-L5, lateral listhesis to the left, progressive degenerative disc disease, and loss of disc height (Figures 1A-1D). Due to the deformity, severe foraminal stenosis, and collapse of the disc space, the patient met indications for transforaminal interbody fusion (TLIF). The patient underwent a left TLIF of L3-S1, revision laminectomy fusion, placement of L5-S1 interbody, and correction of the coronal deformity without complication. The patient had immediate resolution of radicular symptoms during the postoperative period. The patient's postoperative imaging is shown in Figures 2A-2B. The physician author performed the entire five-hour procedure, and the patient tolerated the surgery well without any complications and was subsequently discharged three days later without complications.

On postoperative day (POD) 23, the patient presented with fatigue and characteristic laboratory values of elevated bilirubin and lactate dehydrogenase (LDH) and decreased haptoglobin in her complete blood count (CBC), leading to the diagnosis of anemia. A positive direct Coombs test confirmed the diagnosis of AIHA, and relevant laboratory values are presented in Table 1. The direct Coombs test demonstrated the presence of both polyspecific antihuman globulin (AHG) and IgG AHG, leading to a positive test result. The patient was not given any blood transfusions during surgery with blood loss measuring 100 cc. The patient was given two units of blood on POD 2, one unit on POD 20, and one final unit on POD 23 and demonstrated no improvement as her anemia worsened. The patient also had no history of transfusion reactions in the past. Five days after the positive Coombs test (POD 28), hematology was consulted, and the patient was started on 60 mg of oral prednisone daily. The hematology service concluded that the diagnosis was stress-induced AIHA secondary to recent spinal surgery.

**Table 1:** Patient (female aged 73 years) laboratory values. POD, postoperative day

Variable	POD 23 (Diagnosis)	POD 28	POD 36	POD 39	POD 46	POD 53
Body mass index (kg/m²)	25.43					
Laboratory tests (reference range)						
Lactate dehydrogenase (122-222 U/L)		>500		270	220	171
White blood cell count (3.4 × 10⁹ to 9.6 × 10⁹ L^-1^)			11.2			8.8
Red blood cell count (3.92 × 10¹² to 5.13 × 10¹² L^-1^)			4.32			4.59
Hemoglobin (11.6-15 g/dL)			8.7			9.5
Hematocrit (35.5%-44.9%)			28.9			31.0
Platelet count (157 × 10⁹ to 371 × 10⁹ L^-1^)			322			214
Mean corpuscular volume (78.2-97.9 fL)			66.9			67.5
Mean corpuscular hemoglobin (27-31 pg per cell)			20.1			20.7
Mean corpuscular hemoglobin concentration (32-36 g/dL)			30.1			30.6
Red cell distribution width (12.2%-16.1%)			23.1			21.9
Mean platelet volume (7.5-11.5 fL)			10.5			
Nucleated red blood cell			Slight (Day 8)			
Nucleated red blood cells absolute (0-0.02)			0.41			0.13
Sodium (135-145 mEq/L)				133		133
Potassium 3.5-5.0 mEq/L)				4.5		3.3
Chloride (96-106 mmol/L)				95		100
Carbon dioxide (23-29 mmol/L)				29		28
Blood urea nitrogen (6-20 mg/dL)				17		14
Creatinine (0.5-1.3 mg/dL)				0.80		0.76
Glucose (70-100 mg/dL)				106		100
Calcium (8.5-10.5 mg per deciliter)				9.7		9.6
Total Protein (6.0-8.3 g/dL)				7.1		6.7
Albumin (3.5-5.4 g/dL)				4.8		4.6
Globulin (2.0-3.5 g/dL)				2.0		2.0
Serum glutamic-oxaloacetic transaminase (8-33 U/L)				14		12
Serum glutamate pyruvate transaminase (4-36 U/L)				11		13
Alkaline phosphatase (44-147 IU/L)				70		64
Total bilirubin (0.1-1.2 mg/dL)		2.7		2.2		1.6
Direct bilirubin (<0.3 mg/dL)	0.9					
Direct Coombs test	Positive					
Haptoglobin (42-346 mg/dL)	<10					

Thirteen days after (POD 36) the positive Coombs test, the patient had repeat laboratory studies of a complete metabolic panel (CMP) and CBC with differential. The patient's hemoglobin had improved to 8.7 g/dL at this time, along with the hematocrit (HCT) of 28.9% and platelets (PLT) at 322,000 per microliter. The patient also had a lactate dehydrogenase (LDH) of 270 IU/L, and a total bilirubin of 2.2 mg/dL. The patient's hemoglobin level stabilized, and from a neurosurgery perspective, the patient continued to do well with improved ambulation and no recurrence of radicular lower back pain. Hematology initiated the following taper: 17 days after (POD 40) her positive Coombs test, the prednisone dose was reduced to 40 mg orally every day for two weeks, then to 20 mg orally every day for another two weeks, and finally to 5 mg every week. The patient was tapered off prednisone successfully and returned for weekly laboratory studies while tapering to monitor progress. At the last follow-up visit on POD 99, the patient had no neurosurgical complaints.

## Discussion

AIHA is classified as a type II hypersensitivity reaction. Its etiology can either be idiopathic (primary) or linked to an underlying disease (secondary), both making up approximately 50% of the cases, respectively [1-3]. For secondary AIHA, the most common causes are lymphoproliferative disorders (5%-20%), other autoimmune diseases (1.4%-14%), viral infections (0.02%-20%), or adverse effects of certain medications [4,5]. Many genetic associations with both forms of AIHA exist, with the *CTLA-4* *exon 1* gene being associated with up to 73% of cases and the *TCRG* and *TCRB* genes being associated with up to 50% of cases [5]. AIHA is also separated into two distinct subtypes: warm antibody AIHA and cold agglutinin disease (CAD), based on the temperature at which autoantibodies have an affinity for red blood cells (RBCs) and cause hemolysis. Warm antibody AIHA is the predominant variant, comprising 60%-80% of known cases, and is generally IgG dominant, whereas CAD is IgM dominant [1-7]. Common symptoms of AIHA include fatigue, dark urine, chills, pale color, and shortness of breath. Our patient presented with fatigue as her sole symptom [1-5].

The pathophysiology of warm antibody AIHA involves polyclonal IgG antibodies having the greatest affinity with RBCs at 37 °C, both intravascularly and extravascularly. The opsonized RBCs are then incompletely phagocytosed by macrophages, leading to the formation of spherocytes [8,9]. CD8+ T-cells and natural killer (NK) cells can also contribute to RBC hemolysis through antibody-dependent cell-mediated cytotoxicity (ADCC) [7,9]. The gold standard in the diagnosis of AIHA is the direct agglutination test (DAT) or direct Coombs test, which tests for IgG antibodies as well as C3d. A positive test was noted in this patient along with the characteristic laboratory values such as decreased hemoglobin, elevated bilirubin, elevated LDH, and elevated red cell distribution width (RDW). None of the classic secondary triggers were observed for this patient, as she had no recent viral illness, other autoimmune conditions, or known relevant medications.

Beta thalassemia is an autosomal recessive condition characterized by reduced levels of functional hemoglobin, which is evident from the patient’s baseline hemoglobin of 10 g/dL. Her beta thalassemia minor was evaluated and regarded as stable at the last follow-up five months before surgery. This research shows that there is a positive correlation between beta thalassemia major or intermedia and AIHA [9-12]. A multicenter study spanning 2004-2011 found that about 6.5% of thalassemia patients receiving chronic or intermittent transfusions for treatment had autoantibodies against erythrocytes, significantly higher than the general population [10,13]. Intermittent transfusions were defined as one to seven transfusions within 12 months, while chronic transfusions were defined as more than eight transfusions within 12 months [13]. This study found that increased age also increased the prevalence of autoantibodies [13]. Our patient had no previous transfusions before or during surgery and only received four transfusions after surgery, unrelated to her beta thalassemia minor, which ended on POD 23. There is a definite association between the more severe forms of beta thalassemia and AIHA, but more investigation is needed to determine if a similar relationship is present with beta thalassemia minor, especially after undergoing surgical procedures.

Moon et al. reported a 54-year-old female developing AIHA after developing sepsis from undergoing emergency surgery following blunt abdominal trauma [14]. Sepsis is a rare but possible contributing factor to AIHA [15]. They also stated that the patient received 10 units of packed RBCs and seven units of fresh frozen plasma from the time of admission to the end of the surgery, which could have contributed to the previously mentioned AIHA. However, in our patient, there were no postoperative infections, and our patient received no transfusion during surgery, decreasing the probability of these being contributing factors in our case of AIHA. The similarity of surgery causing increased stress in the body can be noted between this case and our case, as both patients endured long surgeries. It would be interesting to investigate the prevalence of AIHA with postoperative infection and with a length of surgery, respectively.

A secondary form of AIHA, drug-induced immune hemolytic anemia (DIIHA), is elicited by the adverse effects of certain medications [9,16]. There are currently over 150 potentially responsible medications, with the most common being ceftriaxone, piperacillin, and nonsteroidal anti-inflammatory (NSAID) drugs [9]. The exact mechanism is currently not well understood, but it is worth noting that the patient in the case reported by Moon et al. was prescribed piperacillin [14]. They did not report if they tested for any drug antibodies or if piperacillin was administered around the time the AIHA diagnosis was made [14]. It is unclear whether piperacillin after the diagnosis could have affected the disease course of AIHA, and further investigation is warranted. In our patient, none of the common DIIHA drugs were administered before her diagnosis, making this an unlikely cause. This is an even rarer subtype of an already rare condition but should be considered when encountering cases of AIHA on certain medications. Similarities between both cases include a lengthy surgery, which causes increased stress on the body, especially with sepsis in the case of the patient in the study by Moon et al. Caution should also be used when prescribing these drugs to patients if another autoimmune condition exists.

Current treatment guidelines for both primary and secondary warm antibody AIHA are corticosteroids as the first line option, along with the possible addition of rituximab for severe cases [9,16,17,18]. Recommended dosing ranges from 1 to 1.5 mg/kg of body weight for two weeks, followed by a tapering schedule, generally decreasing by 20 mg every two weeks, and then a gradual decrease once 20 mg is reached [9]. Severe cases are defined as hemoglobin below 8 g/dL by the First International Consensus Group [17]. Rituximab is currently considered the second-line treatment if not already utilized with steroids initially; rituximab is also the first-line treatment for refractory AIHA. Sustained remission with steroids is uncommon; only 30% to 40% of patients have sustained remission after one year [9,18]. Our patient is currently on prednisone at 1 mg/kg of body weight on a taper schedule. The patient in the study by Moon et al. was also prescribed a prednisone taper and was discharged 38 days following admission. Our patient appears to be responding well, but caution is warranted for a relapse in the future. Splenectomy is also considered the third line of treatment, with about 70% seeing improvement [9,18].

Surgical procedures are known to cause significant stress on the human body from the increased physiologic processes that occur in the human body following injury and recovery [19]. Stress is a documented trigger of autoimmune diseases, with autoimmune diseases then placing even greater stress on the body as they develop [20]. The duration and invasiveness of surgery are two of the main factors that correlate with increased stress on the body [19]. Our patient underwent an invasive five-hour spinal surgery for radicular pain, with both factors leading to an increased stress response in her body. Inflammation and postoperative pain during the recovery period are also expected after any surgery, also leading to elevated physiological stress on patients creating a favorable environment for autoimmune diseases to develop [19,20].

The healthcare team needs to be cognizant of surgery-induced AIHA, and previous medical records should be investigated for recent surgeries if a patient presents with anemia and obvious etiologies are ruled out. AIHA can be fatal if left untreated; therefore, patient education about the symptoms and follow-up appointments after surgery is imperative.

## Conclusions

This is the first reported case of a neurosurgical patient developing AIHA following routine spinal surgery without surgical complications. The direct Coombs test confirmed the diagnosis along with symptoms at physiological temperature and characteristic laboratory values, indicating a warm antibody AIHA. After a complete hematological workup, the hematology service concluded that AIHA was stress induced by recent spinal surgery. The precise pathophysiological link between surgery and AIHA is unknown, and distinguishing between cause and effect will require additional research. However, this case suggests a possible link between warm antibody AIHA developing after spinal surgery.
